# Implant-Mediated Therapy of Arterial Hypertension

**DOI:** 10.1007/s11906-020-1019-7

**Published:** 2020-02-06

**Authors:** Mortimer Gierthmuehlen, Dennis T. T. Plachta, Josef Zentner

**Affiliations:** 1 Department of Neurosurgery, Ruhr-University Bochum, Universitaetsklinikum Knappschaftskrankenhaus Bochum GmbH, Bochum, Germany; 2 Neuroloop GmbH, Freiburg, Germany; 3 Laboratory for Biomedical Microtechnology, Department of Microsystems Engineering, University of Freiburg-IMTEK, 79110 Freiburg, Germany; 4 Department of Neurosurgery, Faculty of Medicine, University of Freiburg, Freiburg, Germany

**Keywords:** Resistant hypertension, Device- and implant-mediated therapies

## Abstract

**Purpose of Review:**

To give an overview on recent developments in permanent implant-based therapy of resistant hypertension.

**Recent Findings:**

The American Heart Association (AHA) recently updated their guidelines to treat high blood pressure (BP). As elevated BP now is defined as a systolic BP above 120 mmHg, the prevalence of hypertension in the USA has increased from 32% (old definition of hypertension) to 46%. In the past years, device- and implant-mediated therapies have evolved and extensively studied in various patient populations. Despite an initial drawback in a randomized controlled trial (RCT) of bilateral carotid sinus stimulation (CSS), new and less invasive and unilateral systems for baroreflex activation therapy (BAT) with the BAROSTIM NEO® have been developed which show promising results in small non-randomized controlled (RCT) studies. Selective vagal nerve stimulation (VNS) has been successfully evaluated in rodents, but has not yet been tested in humans. A new endovascular approach to reshape the carotid sinus to lower BP (MobiusHD™) has been introduced (baroreflex amplification therapy) with favorable results in non-RCT trials. However, long-term results are not yet available for this treatment option. A specific subgroup of patients, those with indication for a 2-chamber cardiac pacemaker, may benefit from a new stimulation paradigm which reduces the AV latency and therefore limits the filling time of the left ventricle. The most invasive approach for resistant hypertension still is the neuromodulation by deep brain stimulation (DBS), which has been shown to significantly lower BP in single cases.

**Summary:**

Implant-mediated therapy remains a promising approach for the treatment of resistant hypertension. Due to their invasiveness, such treatment options must prove superiority over conventional therapies with regard to safety and efficacy before they can be generally offered to a wider patient population. Overall, BAROSTIM NEO® and MobiusHD™, for which large RCTs will soon be available, are likely to meet those criteria and may represent the first implant-mediated therapeutical options for hypertension, while the use of DBS probably will be reserved for individual cases. The utility of VNS awaits appropriate assessment.

## Introduction

Arterial hypertension is common across the globe. Due to major efforts in prevention and therapy in industrial nations, its global prevalence moved from 29.5%/26.1% (male/female) to 24.1%/20.1% (male/female) in the period between 1975 and 2015. However, due to the increased worldwide population and the increased life expectancy, the actual number of patients with hypertension increased from 594 million in 1975 to 1.13 billion in 2015 [1]. In 2025, 1.56 billion patients are anticipated to have arterial hypertension [2].

Despite medical treatment and new antihypertensive drugs, resistant hypertension—defined as blood pressure (BP) above 140/90 mmHg on at least 3 antihypertensive drugs at optimal doses, typically including a diuretic—is seen in up to 16.9% of hypertensive patients [3]. The American Heart Association (AHA) recently updated their definition of resistant hypertension. It is now defined as (1) above goal BP despite 3 or more BP drugs commonly including a long-acting calcium channel blocker (CCB), a renin–angiotensin system inhibitor (RAS), and a diuretic at maximally tolerated doses and appropriate dosing frequency; (2) exclusion of white coat hypertension by ambulatory blood pressure (ABP) or home blood pressure (BP) monitoring; (3) exclusion of medication non-adherence; and (4) a goal BP defined based on current clinical guidelines. In addition, secondary hypertension has to be excluded, and lifestyle factors should be optimized. The AHA defines elevated BP as a systolic blood pressure (SBP) above 120 mmHg, whereas stage 1 hypertension begins with an SBP of 130 mmHg [4]. As the guidelines recommend initiation of an antihypertensive therapy already in stage 1 hypertension, the number of patients with antihypertensive treatment will even increase in future [1, 5].

Besides medical approaches and destructive techniques like renal denervation, device-mediated therapies have moved to the focus of research within the past years. In this review, we summarize current implant-based therapies for arterial hypertension.

## Baroreflex Activation Therapy

Several sensors in the body register the current arterial (and venous) pressure and report it to the brainstem. In particular, the nucleus of the solitary tract (NTS) receives afferent information about BP and heart rate and initiates countermeasures if the actual BP differs from the set value. This closed-loop system is called baroreflex. The most important pressure sensors are located in the carotid bifurcation (carotid sinus) and the aortic arch (aortic baroreceptors). While the carotid sinus transmits its information via the glossopharyngeal nerve, the aortic baroreceptors use a pathway within the vagal nerve to report the BP to the brainstem.

### Carotid Sinus Stimulation

As the carotid sinus is relatively simple to reach surgically, the electrical stimulation of its baroreceptors with bipolar electrodes on the carotid bifurcation in order to activate the baroreflex and lower the BP has been investigated since 1958 [6]. Bilgutay et al. reported a BP reduction of up to 60 mmHg in hypertensive patients [7, 8] with a simple stimulator with bipolar steel contacts, and first long-term implantations were realized in animal models over several months [9]. As a result of technical difficulties, particularly with regard to connectors and batteries, as well as the ongoing development of new successful antihypertensive drugs, carotid sinus stimulation (CSS) has been forgotten for several decades.

Due to advancements in electronics and biocompatible materials as well as new battery technologies in the late 1990s and early 2000s, baroreflex activation therapy (BAT) generated renewed interest. In 2006, Illig et al. described the Rheos® system as the first implantable carotid sinus stimulator developed by CVRx® (CVRx®, Inc., Minneapolis, MN, USA). Ten patients with therapy-resistant arterial hypertension defined by an office SBP > 160 mmHg despite “appropriate doses of 3 or more antihypertensive medications, including a diuretic” were included in this feasibility trial [10]. Patients with carotid diseases, baroreflex dysfunction, or secondary hypertension were excluded. The Rheos® system consisted of a bilateral carotid sinus stimulation and was able to lower the office SBP by 41 mmHg. In 2010, the results of a larger, non-randomized multicenter study, the DEBuT-HT (Device Based Therapy in Hypertension Trial), were published by Scheffers et al. [11]. Inclusion and exclusion criteria applied were similar to the first trial [10]. Of the 45 patients enrolled, 37 completed the 3-month, 26 the 12-month, and 17 the 24-month follow-ups. Office systolic/diastolic blood pressure (SBP/DBP) was reduced by 21/12 mmHg after 3 months, 30/20 mmHg after 12 months, and 33/22 mmHg after 24 months. The procedure was well tolerated by the patients with a serious adverse event (SAE) rate comparable to carotid surgery. Due to protocol reasons (which were not further described in the publication), 3 patients were excluded from SAE analysis. Of the remaining 42 subjects, 7 had a procedure-related SAE of which one, a possibly drug-induced angioneurotic edema, was fatal and 3 devices had to be removed due to infection. One perioperative stroke and one hypoglossal paresis were encountered. One device-related SAE was observed due to movement of the implanted IPG which had to be repositioned in a second surgery.

Within the Rheos Pivotal Trial, the CSS system has been implanted in 322 patients with resistant hypertension. Group A had an active stimulation for the first 6 months; Group B started with a deactivated device which was then activated after 6 months. Of the 265 patients evaluated, 81% had an office BP decrease of at least 10 mmHg after 12 months with the average reduction of up to 26 mmHg (± 30 mmHg) after 6 months and 35 mmHg (± 28 mmHg) after 12 months [12]. There were no significant differences between group A and group B. Procedural SAE occurred in 25.5% including 25 nerve injuries, 7 respiratory complications, 7 wound infections, and 13 not further defined “general surgical complications.” Device-related SAE occurred in 12.8%, of which only hypertension-related strokes with 6 cases were further explained. So far, this was the only large randomized controlled trial of a CVRx system. Overall, the study failed to achieve two primary endpoints: acute responders after 6 months (no significant difference between the stimulated and non-stimulated groups) and primary safety endpoints (procedural risk higher than pacemaker implantation). Bakris et al. re-evaluated 276 of the 322 implanted patients after an average time of 28 (± 9) months [13]. Of them, 76% were “clinical responders” and had an average office BP reduction of 35/16 mmHg (SBP/DBP), and 10% were intermediate responders with an average reduction of 19/10 mmHg (SBP/DBP).

In 2017, the 6-year results of the Rheos Feasibility Trial, the DEBuT-HT Trial, and the Rheos Pivotal Trial were published by de Leeuw [14]. In all patients included in these studies, the Rheos System had been implanted bilaterally. Of 383 implanted patients, 142 terminated BAT during the follow-up phase, 34 of them during the first year of implantation, while 14 patients did not qualify for battery exchange (average battery lifetime was 1.5 years) due to insufficient response. The median implantation time was 5 years. Office SBP dropped significantly from an average of 179 ± 24 to 144 ± 28 mmHg, while DBP significantly decreased from 103 ± 16 to 85 ± 18 mmHg. The highest drop in BP was found in patients with congestive heart failure (mean reduction of 46/24 mmHg compared with mean reduction of 32/16 mmHg throughout all participants). Patients with isolated systolic hypertension had the lowest BP reduction with a mean of 23 ± 7 mmHg. In 27% of the patients, the number of drugs could be reduced from an average of 6 to 3, while in 39%, the number of antihypertensive drugs had to be increased from an average of 5 to 7. During the entire follow-up time, 335 serious adverse events were observed in 111 patients. Of these events, 26 (7.8%) were device or procedure related and occurred in 23 patients (7%). Limitations of this follow-up study related to the integration of 2 non-randomized studies and the fact that only office BP was analyzed.

Due to the high periprocedural complication rate of the bilateral implantation as mentioned, a new device—the BAROSTIM NEO®—was developed. It featured a smaller unilateral electrode, which has been implanted in 90% of the patients on the right side. This new device was first investigated in a non-randomized trial in 30 patients [15]. The periprocedural complication rate dropped to 10%. The mean reduction in systolic office BP after 6 months was 26 mmHg (± 4.4 mmHg). Battery lifetime was calculated to reach 2.8 ± 1.4 years due to reduced energy consumption of unilateral stimulation. These findings of an efficient unilateral stimulation were supported by a post hoc analysis of both groups of the Rheos Pivotal Trial [16]. According to this report, 215 of the 295 enrolled patients had only unilateral stimulation of their bilateral electrodes, 127 on the right side and 88 on the left side. In the unilateral group, the office SBP dropped from 178 ± 23 to 146 ± 30 mmHg. The 80 bilaterally implanted patients had an office SBP reduction from 178 ± 23 to 155 ± 31 mmHg. Therefore, de Leeuw postulated a unilateral right-sided dominance in baroreflex activation and a possibly more effective BAT with electrodes being implanted on the right side. Heusser et al. analyzed 18 patients with unilateral CSS. Initially, the SBP was lowered by 16.9 ± 15 mmHg. Due to discomfort during stimulation, the intensity had to be reduced in 12 of these 18 patients, which resulted in a decreased efficiency with a decrease in SBP of only 6.3 ± 7 mmHg [17]. It was discussed whether modified electrodes might focus the stimulation to a specific site, thus reducing side effects.

An open-label and single-arm evaluation of 17 unilaterally implanted patients reported a mean reduction of the office SBP by 26 mmHg and of the DBP by 13 mmHg at a mean follow-up of 16.5 months [18]. After acute deactivation of the device, BP increased by 11/5 mmHg (SBP/DBP). Similar results were found by Beige et al. in a randomized and blinded crossover study including 16 patients in whom the BAROSTIM NEO® had been implanted 2.7 ± 1.1 years before. The study had a crossover design: 8 of those patients started with the device being deactivated for 4 weeks and then activated, while the other 8 participants started with an activated device which was turned off after 4 weeks. Four patients dropped out. The results of 12 patients were analyzed. Both office BP and ABP increased significantly during deactivation by a mean of 10 mmHg after 4 weeks, but never reached the level before implantation of the BAT device. As the investigators initially expected an increase of more than 35 mmHg, the study did not reach its primary endpoint [19]. The prolonged reduction of BP after baroreflex stimulation is in accordance with the observation of Plachta et al. who demonstrated a longer lasting BP-lowering effect of intermittent vagal nerve stimulation (VNS) [20•].

In 2016, Wallbach et al. published results of a prospective non-randomized trial. Of 51 enrolled patients who were implanted with BAROSTIM NEO®, 44 were analyzed. Office BP was reduced from 171 ± 24 mmHg/91 ± 18 mmHg (SBP/DBP) to 151 ± 26 mmHg/82 ± 17 mmHg (SBP/DBP) under stimulation. However, 24-h ambulatory BP measurements (ABPM) only showed a reduction from 148 ± 17 mmHg/82 ± 13 mmHg (SBP/DBP) to 140 ± 23 mmHg/77 ± 15 mmHg (SBP/DBP). Wallbach et al. [21] described 23% minor procedure–related complications, one procedure-related contralateral stroke (2%), and 5% device-related complications.

As BAT modulates the autonomic nervous system, it also seems to be beneficial for patients with NYHA-III heart failure [22, 23]. Therefore, a randomized, open-label trial “BeAT-HF” (NCT02627196) with 1200 estimated participants is currently carried out. Patients with receiving BAT based on BAROSTIM NEO® are compared with patients receiving optimal guideline-directed medical therapy (GDMT). Another observational case-control trial (“Evaluation of Baroreflex Activation Therapy in Patients With Advanced Heart Failure,” NCT03230643) is active, but not yet recruiting. This study plans to involve 200 patients with heart failure who receive a BAROSTIM NEO® device.

As hypertension is frequently associated with renal impairment and BAT seems to have a nephroprotective effect [24], Beige et al. [25] studied whether patients with end-stage renal failure benefit from BAT. In 6 patients, who were enrolled in other prospective BAT studies with BAROSTIM NEO®, the office SBP after 12 months decreased significantly from 194 ±28 to 137 ± 16 mmHg. The office DBP decreased from 97 ± 19 to 73 ± 17 mmHg. ABP also showed a decrease in SBP and DBP from 167 ±30 mmHg (SBP) and 94 ±24 mmHg (DBP) to 134 ±27 mmHg (SBP) and 79 ±22 mmHg (DBP). However, due to the limited number of patients, this decrease both for SBP and DBP was not significant [25].

In 2016, Wallbach et al. published a non-randomized retrospective investigation of 28 patients who had uncontrolled hypertension despite previous renal denervation at least 5 months before [26]. After 6 and 12 months, office SBP was significantly reduced by 18/21 mmHg, while office DBP showed a non-significant decrease of 5/5 mmHg. The ABP measurements showed an unaltered BP after 6 months and a significant reduction of 14 ± 23 mmHg after 12 months. The 24-month results of this study including further patients were published in 2019 [27••]. In 60 patients, office BP dropped significantly from initial 172 ± 25 mmHg/90 ± 17 mmHg to 145 ± 24 mmHg/81 ± 17 mmHg (SBP/DBP) after 24 months. ABP decreased significantly from initial 150 ± 16 (SBP) and 80 ± 12 mmHg (DBP) to 140 ± 17 mmHg (SBP) and 76 ± 12 mmHg (DBP). Patients with prior renal denervation (*n* = 19) had a tendency towards a less pronounced drop in ABP compared with patients without prior renal denervation. In summary, 50% of the patients had a significant reduction in ABP of more than 5 mmHg (= responders).

In 2015, the “European Clinical Consensus Conference” formulated some prerequisites for future clinical trials on resistant hypertension [28]. As non-adherence to antihypertensive medication is one of the main reasons for inadequately controlled hypertension [29], investigators should monitor drug adherence prior and during the trials. In addition, ABP monitoring was thought to be mandatory to avoid the white coat effect. In contrast to the usual practice with device trials and especially with destructive procedures such as renal denervation, a real sham group including operated but non-treated participants is considered not to be acceptable for ethical reasons and therefore not mandatory for future trials of resistant hypertension. Crossover designs in which one group receives an implant which is activated after a delay seem to be an appropriate compromise.

National and international committees have analyzed BAT as a possible option for resistant hypertension. The ESC/ESH guidelines see a high potential in BAT but demand further randomized controlled trials (RCTs) with the unilateral BAROSTIM NEO® system before any recommendations can be made [30]. The German BAT Consensus Group considers BAT as a potential therapeutic option for resistant hypertension, but recommends that patients should be embedded in clinical trials in specialized centers until further RCTs with unilateral stimulation are available [31]. The “Norwegian Institute of Public Health” criticized the lack of evidence to demonstrate efficiency and cost-effectiveness [32]. New AHA guidelines include BAT as a potential option which needs further evaluation by RCTs [33, 34].

The BAROSTIM NEO® Pivotal Trial (NCT01679132) was designed as a single-group open-label trial including 10 participants. However, this trial was suspended, since the company focused on other studies [35]. Currently, two large randomized controlled trials for baroreceptor activation therapy are carried out, investigating the unilateral BAROSTIM NEO® System. In Finland, the Nordic BAT study (NCT02572024) is recruiting a total of 100 patients with systemic ambulatory BP of 145 mmHg or more and/or diastolic ambulatory BP of 95 mmHg or more. These patients receive a carotid sinus stimulator and are assigned to the “BAT-group” with activated stimulator or the “Placebo-group” with deactivated stimulator. The “Economic Evaluation of Baroreceptor STIMulation for the Treatment of Resistant HyperTensioN (ESTIM-rHTN, NCT02364310)” trial is a multicenter study in France including 128 participants with resistant hypertension. It randomly enrolls patients in either of two groups: “BAT-group” and “Best Medical Care-Group.” In addition, the “UK Registry for Baroreflex Activation Therapy (UK-BAT, NCT03730519)” will include 25 participants with either resistant hypertension (> 150 mmHg) or volatile BP, but is not yet enrolling. However, this will not be a randomized controlled trial.

In summary, BAT is a technique originating in the 1960s of the twentieth century which has been developed to a long-term stable therapeutical option for resistant hypertension. However, RCTs are still needed before it can be recommended in daily routine (and reimbursed by healthcare insurances). As some RCTs are being conducted (but not by CVRx®), interesting and clarifying results shall be available in a few years.

Baroreflex activation through an endovascular approach was investigated in the “Acute Carotid Sinus Endovascular Stimulation II” by Medtronic® (NCT01458483). This non-randomized, single-group study was completed with 9 participants in October 2012, but results have not been published yet.

### Baroreflex Amplification Therapy

Baroreflex amplification via an endovascular stent (Mobius HD®, Vascular Dynamics, Mountain View, CA, USA) has recently been proposed as an antihypertensive intervention. The group first published a computer simulation that an endovascular carotid stent could increase circumferential and longitudinal stress in the carotid sinus by 2.5% and 7.5%, respectively, and therefore activate the baroreflex [36]. A canine model demonstrated a BP reduction of 50/30 mmHg (SBP/DBP) for 6 h, but the stent would not stay patent due to the limited diameter of the dog’s carotid sinus [37••]. Based on these findings, the “Controlling and Lowering Blood Pressure with The MobiusHD™” study (CALM-FIM_EUR, NCT01911897) was initiated constituting an open-label safety and efficacy, first-in-man (FIM) multicenter study. A total of 30 participants with resistant hypertension on 3 sufficiently dosed medications with stable hypertension over the last 30 days were enrolled. A minimum adherence to medical therapy of 80% was ensured by self-reports of the patients. In total, 19 patients received the stent on the right and 11 on the left side. Serious adverse events (SAE) were observed in 20% including leg claudication due to a dislocated femoral closure device and two focal neurological deficits interpreted as minor strokes. Initial office BP reduction was significant with 38/23 mmHg (SBP/DBP) and remained stable until 6 months after implantation, still significant with 24/12 mmHg (SBP/DBP). ABP reduction was also significant after 3 months (15/8 mmHg SBP/DBP) and after 6 months (21/12 mmHg SBP/DBP). Initially, 73% of the patients had a BP reduction of at least 10 mmHg in office BP and 5 mmHg in ABP. The responder rate increased to 83% after 6 months. Limitations of this study refer to the limited number of participants as a FIM study, the unblinded study design, and the fact that medical adherence was only monitored by a self-reported diary of the patients and not by urine analysis. In addition, long-term effects on BP should be evaluated as an adaption of the baroreceptors to elevated BP (which is probably mimicked by an elevated wall stress) [38].

The US version of the study (CALM-FIM_US, NCT01831895) with similar inclusion criteria as used for the CALM-FIM_EUR study has not yet been published. The ongoing CALM-DIEM single-group assignment trial (NCT02827032) has been designed to define efficiency markers in 200 participants. The CALM-START study (NCT02804087) has a randomized, blinded, and sham-controlled design and is currently enrolling 110 participants. The CALM-2 study (NCT03179800) will include 300 patients in a randomized crossover assignment and sham implantation. In the two latter trials, it will be interesting how a sham intervention is accomplished in such an interventional study design. Certainly, the results of these studies and further long-term analysis will elucidate whether MobiusHD is a promising approach for the treatment of resistant hypertension.

In summary, baroreflex amplification therapy is a young endovascular approach against hypertension, which—if the effect is sustainable—can be very elegant. Larger RCTs are necessary to evaluate the efficacy and the SAE profile. Especially in the presence of atherosclerosis, which is frequently observed in hypertensive patients, endovascular approaches harbor the risk of stroke, as already seen in this study.

### Vagal Nerve Stimulation

In addition to the sensors in the carotid sinus, the BP is also monitored by baroreceptors in the right subclavian artery and aortic arch. The right vagal nerve innervates the baroreceptors of subclavian artery, the left vagal nerve the baroreceptors of the aortic arch [39–41]. While the right vagus is predominantly associated with the sinus node, the left vagal nerve is linked to the AV node with less influence on the heart rate [42]. Owing to this observation, only left-sided VNS is approved for the treatment of epilepsy [43], although single studies have demonstrated safety and efficacy also for right-sided VNS [44].

In some species such as the rat, the nerve fibers transmitting the BP information to the brainstem run as a separate strand (aortic depressor nerve, ADN) parallel to the vagal nerve [45]. Isolated bipolar stimulation of the ADN is known to activate the baroreflex and lower the BP [46], and the ADN shows a neural activity which is directly correlated with the arterial blood pressure [47]. In the sheep [48], pig [41], and dog [49], the ADN enters the vagal nerve very caudally and leaves it below the superior laryngeal nerve for just a short distance unit it reenters the vagal nerve. Although the ADN cannot be identified as a separate strand in humans, neuroanatomical studies [50] and the known vagal termination in the nucleus of the solitary tract (NTS) [40] suggest a similar somatotopy.

Vagal nerve stimulation (VNS) has first been investigated in the treatment of epilepsy in 1990 [51]. Its efficiency to reduce seizure frequency has been demonstrated in many observational studies as well as in large randomized trials over the last decades [52, 53]. The antiepileptic stimulation is usually adapted to the on/off paradigm of DeGiorgio with 30-s-long stimulation at 30 Hz, 0.25 mA, and a pulse width of 500 μs, followed by a pause of 5 min [54]. Clinical trials found no influence of the DeGiorgio stimulation modality on BP, ECG, and heart rate [55].

In preclinical studies, VNS has been found to show a cardioprotective effect in artificially induced myocardial infarction [56], and to improve efficacy of the cardiac performance [57] by reduction of sympathetic tone. Therefore, VNS was investigated for the treatment of heart failure (HF). First, randomized studies with 96 and 60 participants, respectively (NECTAR-HF [58] and ANTHEM-HF [59]), showed a significant improvement mostly in the subjective assessments of NYHA functional class and quality of life score. However, Hawthorne and placebo effects could have played a role for these results. The largest randomized trial with 730 participants (INOVATE-HF [60]) was prematurely aborted by the Steering Committee on the recommendation of the Data and Safety Monitoring Board, since the interim analysis did not show any significant differences between the treatment and the control groups.

The rationale to use VNS for the treatment of hypertension is to selectively stimulate those baroreceptor fibers travelling in the left vagal nerve while avoiding co-stimulation of other vagal sections. Such selectivity can be achieved with new multi-contact cuff electrodes (MCE) made of polyimide and harboring over a dozen of contacts [61, 62]. In preclinical studies, it has been shown that the BP in rats could be reduced up to 40% of the baselines without causing significant bradycardia [63]. Interference with commonly used antihypertensive medication seemed to be minimal [64–66]. This method of activating the baroreflex also seems to keep the BP low even without constant stimulation, but synchronized to the electrocardiogram (ECG) [20•]. However, experience with chronic implantation of the device in a larger animal model as well as long-term results is not yet available. Furthermore, first-in-man studies are necessary to evaluate whether the effects observed in animal experiments can be confirmed in humans.

## Deep Brain Stimulation

Deep brain stimulation (DBS) has been used in humans to treat central nervous diseases like movement disorders and psychiatric conditions since the early 1990s [67]. However, already in 1935, Kabat et al. were able to influence BP in cats by stimulating certain areas in the periaqueductal gray (PAG). Inui et al. confirmed that electrical and chemical stimulation of the PAG alters the baroreflex and the BP, respectively, in rats [68]. An antihypertensive effect of DBS in humans was first described in a study by Green et al. in 2005. Of 15 patients who received DBS in the PAG for neuropathic pain, 7 patients experienced a significant reduction of BP by 14.2 ± 3.6 mmHg/4.9 ± 2.9 mmHg (SBP/DBP) on the average when the ventral PAG was stimulated. Contrarily, 6 patients showed a significant increase in BP of 16.73 ± 5.9 mmHg/4.9 ± 2.8 mmHg (SBP/DBP) when the dorsal PAG was activated [69].

These findings were confirmed in 2007 reporting the case of a 61-year-old male patient. During DBS implantation of a PAG electrode for neuropathic pain, stimulation of its ventral portion reduced BP from 157.4/87.6 to 132.4/79.2 mmHg (SBP/DBP). Contrarily, stimulation of the dorsal PAG increased SBP to a mean of 179 mmHg [70]. Patel et al. reported a patient who received DBS for post-stroke neuropathic pain. The preoperative office BP under quadruple antihypertensive therapy ranged from 134/72 to 153/87 mmHg. After activation of DBS, the BP decreased to 80/53 mmHg and stabilized at 118/70 mmHg with reduced medication after 33 months [71]. Remarkably, the reduction of BP was independent from pain relief and persisted when the pain returned to preoperative levels 4 months after surgery. Moreover, a mean BP reduction of 12.6/11.0 mmHg (ambulatory SBP/DBP) was observed 8 months after left-sided ventral PAG DBS in a case reported by Pereira [72].

O’Callaghan et al. implanted a DBS system in a patient who previously had renal denervation and a CSS device. Despite these approaches and a regimen of 8 antihypertensive drugs, he continuously showed excessive office BP up to 320/150 mmHg [73]. ABP could mostly not be measured as the ABP monitoring systems do not allow BP measurements exceeding 240 mmHg. Immediately after implantation of the device with the stimulator still being turned off, the BP fell to 125/68 mmHg, possibly due to the irritative effect of the electrode on the periaqueductal gray, and rose to 205/130 mmHg after 72 h. After activation of the DBS 4 days after surgery, the ABP decreased to 170/109 mmHg during the day and 119/77 mmHg during the night. As the patient complaint of lethargy, the antihypertensive medication was reduced. After 2 years, 24-h measurements showed a constant decrease of ABP to 225/142 mmHg during daytime and 155/102 mmHg during nighttime.

The non-randomized, single-group interventional study “Treatment of Pain and Autonomic Dysreflexia in Spinal Cord Injury with Deep Brain Stimulation” (NCT02006433) was completed in September 2017. It included 12 participants who received DBS in the PAG/PVG region for neuropathic pain, with one endpoint to assess the influence of DBS on arterial BP. However, the results have not yet been published. The abovementioned studies focused on the ventral PAG as the DBS target. In one publication, a mean SBP decrease of 15 mmHg was found in 4 patients during stimulation of the rostral subcallosal neocortex (Brodmann area 25) for epilepsy [74]. Other deep brain locations are being discussed as theoretically possible targets to manipulate BP. However, further investigations are necessary to identify the areas which have to stimulated to reliably decrease the BP [75].

## Pacemaker-Mediated Reduction of Blood Pressure

The variation of the atrioventricular delay can alter the BP due to an increased or reduced filling of the left ventricle [76]. The Moderato-HTN-trial, a first single-arm, multicenter, and non-randomized safety and efficacy study, enrolled 35 patients in whom the implantation of a dual-chamber pacemaker due to persistent hypertension was indicated [77••]. A special program with accelerated AV conduction time was activated in 27 patients, leading to a reduction of office SBP by 16 ± 15 mmHg and ABP by 10 ± 13 mmHg after 3 months. Especially patients with isolated hypertension responded well to this treatment. However, detailed information on analytic methods and data has not yet been given. The limitations of this trial refer to its non-randomized design, including only a small number of a subgroup of patients, i.e., those who need a pacemaker. In addition, this study was not blinded, and the follow-up time was short. A compensating sympathetic activation was not seen in this first trial, and only long-term results can be expected to confirm sustainable BP decrease over time. To overcome these limitations, two double-blinded studies are planned: “Moderato System: A Double-Blind Randomized Trial Ver 1.1” (170 patients, NCT02837445) and “Moderato System in Patients With Hypertension” (190 patients, NCT03757377).

## Conclusion

Today, BAT constitutes the most advanced surgical option for patients with resistant hypertension. While the first iteration of the CVRx-System Rheos® did not meet endpoints in the large RCT, the second-generation BAROSTIM NEO® showed promising results in smaller studies. Yet, a large RCT is needed to demonstrate a significant benefit along with safe application. Additionally, cost-effectiveness calculations comparing expenses between BAT and conservative medical treatment are necessary. So far, BAT is only recommended in specialized centers and within clinical trials. The endovascular baroreceptor reshaping approach seems to be very elegant. However, long-term results of the CALM studies including safety aspects have to be expected. In addition, the efficacy of VNS awaits appropriate clinical assessment. The use of DBS will be limited due to the major invasiveness of this procedure.

## Abbreviations

ABP: Ambulatory blood pressure
ADN: Aortic depressor nerve
AHA: American Heart Association
BAT: Baroreflex activation therapy
BP: Blood pressure
CSS: Carotic sinus stimulation
DBP: Diastolic blood pressure
ECG: Electrocardiogram
GDMT: Guideline-directed medical therapy
FIM: First in man
HF: Heart failure
NTS: Nucleus of the solitary tract
NYHA: New York Heart Association
PAG: Periaqueductal gray
PVG: Periventricular gray
RCT: Randomized controlled trial
SAE: Serious adverse event
SBP: Systolic blood pressure
